# Surveillance After Thoracic Aortic Surgery Yields to Low Number of Reinterventions: A Retrospective Cohort Study

**DOI:** 10.1093/icvts/ivag239

**Published:** 2026-08-26

**Authors:** Henri-Jussi Kurvinen, Ari Mennander, Mikko Uimonen

**Affiliations:** Faculty of Medicine and Health Technology, Tampere University, Tampere, 33520, Finland; Faculty of Medicine and Health Technology, Tampere University, Tampere, 33520, Finland; Heart Hospital, Tampere University Hospital, Wellbeing Services County of Tampere, Tampere, 33520, Finland; Faculty of Medicine and Health Technology, Tampere University, Tampere, 33520, Finland; Heart Hospital, Tampere University Hospital, Wellbeing Services County of Tampere, Tampere, 33520, Finland

**Keywords:** thoracic aneurysm, aortic surgery, follow-up

## Abstract

**Aim:**

Postoperative imaging surveillance after thoracic aortic surgery is widely recommended, but its clinical yield, efficiency, and optimal targeting remain poorly defined. We aimed to quantify imaging intensity, determine the procedural yield of surveillance imaging, and assess whether follow-up strategies could be better individualized.

**Methods:**

We conducted a retrospective single-centre cohort study of patients undergoing thoracic aortic surgery between 2010 and 2024. Postoperative imaging examinations were reviewed and linked with subsequent treatment decisions. Imaging findings, reinterventions, timing of events, detection context, and follow-up patterns were analysed.

**Results:**

The cohort included 380 patients with 1757 postoperative imaging examinations, of which 1074 were classified as surveillance aortic imaging performed ≥30 days after surgery. Overall, 42 examinations led to reintervention in 29 patients (7.6%), including 13 examinations leading to aortic reoperation in 12 patients. Among surveillance aortic examinations, 41 (3.8%) led to reintervention and 16 (1.5%) met treatment thresholds without immediate intervention, yielding a combined directly or potentially actionable finding rate of 5.3%. This corresponded to 26.2 surveillance examinations per reintervention and 82.6 per aortic reoperation. Cumulative incidences of first reintervention were 2.8% at 1 year, 5.9% at 3 years, and 8.3% at 5 years. Most reinterventions were identified during elective surveillance imaging (71.4%). Despite more frequent imaging among emergency patients, reintervention risk and imaging yield were similar to those of non-emergency patients.

**Conclusions:**

Postoperative imaging surveillance after thoracic aortic surgery was associated with substantial imaging burden but low immediate procedural yield, with clinically relevant findings concentrated in a minority of patients. These findings support the need to better define patient-specific risk and imaging efficiency but do not establish that less intensive surveillance would be safe without prospective validation.

## Graphical abstract

**Figure:**
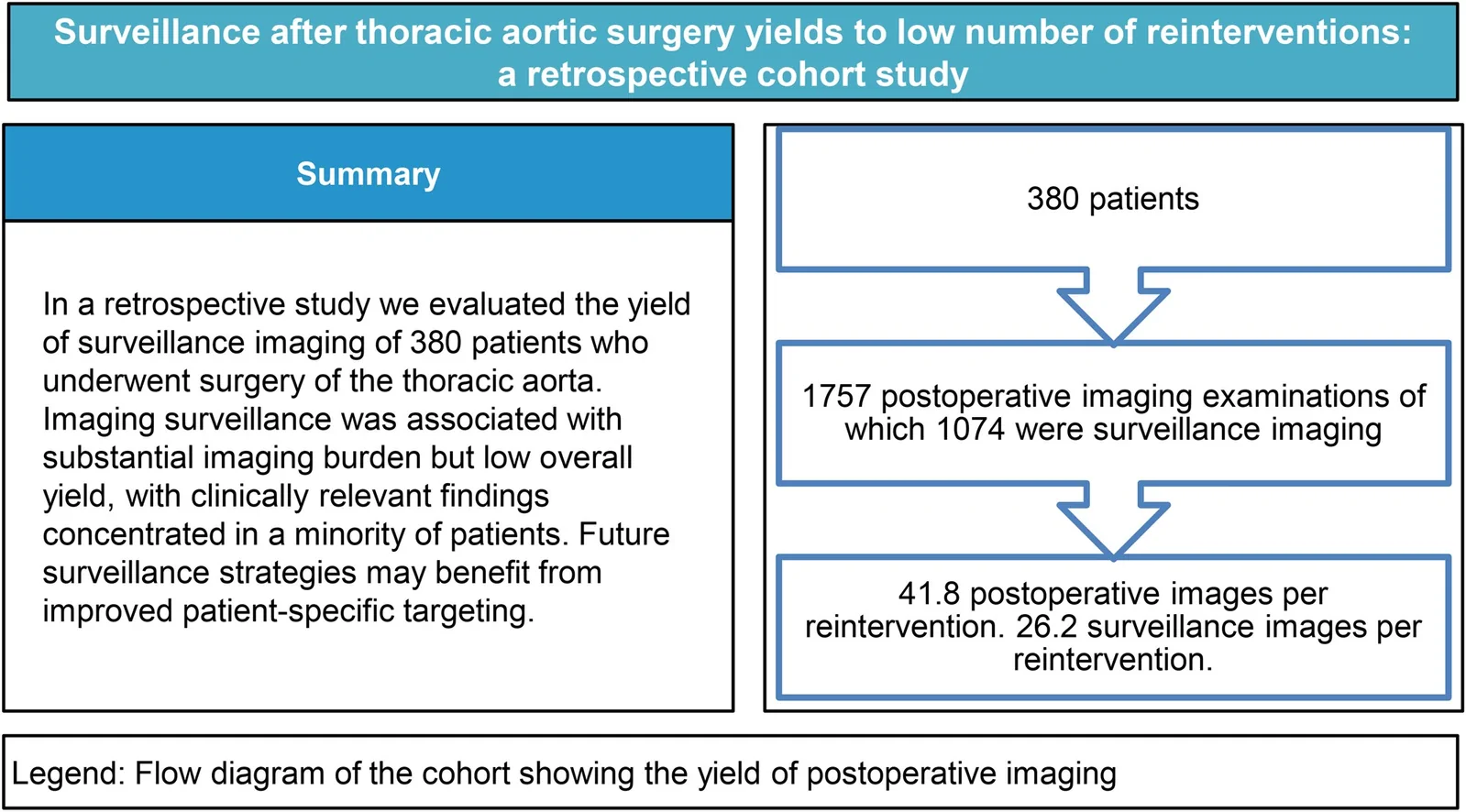

## INTRODUCTION

Postoperative imaging surveillance is a central component of long-term management after thoracic aortic repair to detect postoperative complications and progressive aortic dilatation.^1^^,^^2^ Guideline recommendations advocate repeated imaging after surgery, but the suggested intervals vary and are largely based on limited observational data and expert consensus rather than high-quality comparative evidence. The American Heart Association/American College of Cardiology recommend imaging within the first postoperative year and thereafter at 5-year intervals in patients without residual aortopathy, with more frequent imaging if abnormalities are present.^1^ The European Society of Cardiology recommends a more intensive early surveillance strategy, including imaging at 1 month, 6-12 months, and 2 years after surgery, followed by periodic long-term follow-up.^2^ European Association for Cardio-Thoracic Surgery/Society of Thoracic Surgeons (EACTS/STS) guidelines recommend individual risk-based surveillance due to the lack of data on any surveillance program being superior.^3^ More frequent imaging is generally advised after endovascular repair due to higher rates of device-related complications.^1^^,^^2^^,^^4–6^

In this study, we evaluated postoperative imaging after thoracic aortic surgery in a real-world cohort. Specifically, we aimed to quantify imaging intensity, determine the yield of surveillance imaging in detecting reinterventions, and assess the temporal distribution and context of clinically relevant findings. We hypothesized that the yield of routine surveillance imaging is low and unevenly distributed, suggesting that follow-up protocols could be optimized through better risk-based targeting.

## METHODS

### Study design and population

This retrospective cohort study included all consecutive adult (≥18 years old) patients who underwent open aortic surgery as a treatment of thoracic aortic disease encompassing procedures involving the ascending or descending thoracic aorta or the aortic arch at a single tertiary referral centre between 2010 and 2024. Patients were identified through the institutional patient and procedural registry. Indications for surgery included a broad spectrum of thoracic aortic pathologies, including aneurysmal disease, dissection, and other aortic conditions requiring operative management. All included patients were followed from the time of index aortic surgery. Follow-up extended until death or the end of the study period. Patients were followed through regional healthcare records covering the entire catchment area. Complete follow-up was available for all patients within the region.

### Imaging exposure and surveillance strategy

Postoperative imaging surveillance of the thoracic aorta was the main focus of the study. All cross-sectional imaging studies of the thoracic region and the aorta performed after surgery were systematically reviewed, including computed tomography (CT), magnetic resonance imaging (MRI), and positron emission tomography combined with CT (PET-CT). Imaging examinations were classified according to their clinical context. Elective surveillance imaging was defined as planned follow-up imaging performed as part of routine postoperative monitoring. Non-elective imaging included examinations performed due to symptoms, clinical deterioration, or other urgent indications.

In routine clinical practice, follow-up imaging examinations were first interpreted by radiologists experienced in aortic imaging. The findings were subsequently reviewed by the responsible aortic surgeon, who interpreted the imaging results in relation to the patient’s clinical status, operative history, comorbidities, age, and overall suitability for further intervention. The final management decision, including continued surveillance, discontinuation of follow-up, additional imaging, or reintervention, was made by the responsible aortic surgeon based on this overall clinical assessment. Imaging examinations were categorized according to the radiological findings and the subsequent clinical management decision, including stable findings, progression meeting treatment thresholds, discontinuation of surveillance, and findings directly leading to reintervention. Reintervention was defined as any open surgical or endovascular aortic procedure performed after postoperative imaging and linked to the imaging finding and subsequent clinical decision-making. The analysis did not distinguish between staged/planned and unexpected/unplanned reinterventions, because the original operative intent and long-term staged repair strategy could not be determined reliably and uniformly from the retrospective data.

### Outcomes

The primary outcome was imaging-associated reintervention, defined as any open surgical or endovascular aortic or branch vessel reintervention performed after postoperative imaging and linked to the imaging finding and subsequent clinical decision-making. The main secondary outcome was aortic reoperation. Imaging yield was defined as the clinical output of postoperative imaging surveillance, expressed as the proportion of imaging examinations leading to reintervention or aortic reoperation, or showing findings that met treatment thresholds without immediate intervention. Yield was also expressed as the number of imaging examinations required to identify 1 such event. Timing of reintervention, detection context, imaging intensity, surveillance discontinuation, death during follow-up, and emergency versus non-emergency subgroup findings were analysed descriptively.

### Data collection

For each imaging examination, the imaging modality, timing relative to surgery, clinical indication, and key findings were recorded. In addition, subsequent management decisions were documented, including whether imaging findings resulted in continued surveillance, treatment escalation, or reintervention. At the patient level, baseline clinical characteristics, operative details, follow-up duration, occurrence and timing of reinterventions, and survival status were collected. Information on discontinuation of imaging surveillance and the underlying reasons (eg, clinical decision, patient condition, or death) was also obtained.

### Statistical analysis

Analyses were conducted at both the imaging and patient levels. Imaging-level analyses used individual imaging examinations as the unit of analysis and were primarily descriptive, whereas patient-level analyses used each patient once. Continuous variables are reported as medians with interquartile ranges, and categorical variables as counts and percentages.

The primary outcome was any imaging-associated reintervention, defined as a surgical or endovascular procedure following an imaging examination. Aortic reoperation was analysed as a secondary outcome. Imaging yield was quantified as the proportion of examinations leading to reintervention or aortic reoperation and as the number of imaging examinations required to detect 1 event. Yield was calculated both for all postoperative imaging and separately for surveillance aortic imaging. Reintervention rates were also expressed per patient-year of follow-up.

Time-to-event analyses were performed from the date of index surgery to first reintervention. Patients without reintervention were censored at death or at the end of follow-up. Kaplan-Meier methods were used to describe freedom from first reintervention. Because death may preclude later reintervention, death without prior reintervention was summarized descriptively as a competing outcome. No competing-risk regression model was fitted because of the limited number of reintervention events.

Comparisons between patients with and without reintervention were performed descriptively. Exploratory logistic regression models were used to assess associations between selected clinical factors and patient-level reintervention. Variables were selected a priori based on clinical relevance. The main clinical model included age at surgery, sex, and EuroSCORE II. A second exploratory model included age at surgery, EuroSCORE II, and the number of surveillance-phase imaging examinations to assess the association between imaging intensity and reintervention. Odds ratios are reported with 95% CIs. These models were considered exploratory and were not intended for risk prediction.

A prespecified subgroup analysis compared patients undergoing emergency versus non-emergency index procedures based on procedural urgency. Differences between groups were assessed at the patient and imaging levels, including reintervention rates, imaging intensity, temporal distribution of events, and detection context. Categorical variables were compared using Fisher’s exact test and continuous variables using the Wilcoxon rank-sum test. An exploratory logistic regression model assessed the association between emergency status and reintervention adjusted for age, sex, and EuroSCORE II.

Outcome and follow-up data were available for all patients within the regional healthcare system. Missing baseline covariate values were not imputed; regression analyses were performed as complete-case analyses. All analyses were conducted using R (R Foundation for Statistical Computing).

### Ethical considerations

The study was approved by the institutional review board of Wellbeing Services County of Pirkanmaa on October 23, 2025 in accordance with local regulations (approval number R25843X). Due to the retrospective nature of the study and use of existing clinical data, the requirement for individual informed consent or ethics committee approval was waived.

## RESULTS

The study cohort included 380 patients with a total of 1757 postoperative imaging examinations (**Figure 1**, **Table 1**). Of these, 1475 (83.9%) were classifiable according to predefined imaging categories, most commonly reflecting stable aortic status not meeting threshold criteria (1027 examinations, 58.5%). Imaging leading directly to reintervention accounted for 42 examinations (2.4%).

**Figure 1.**
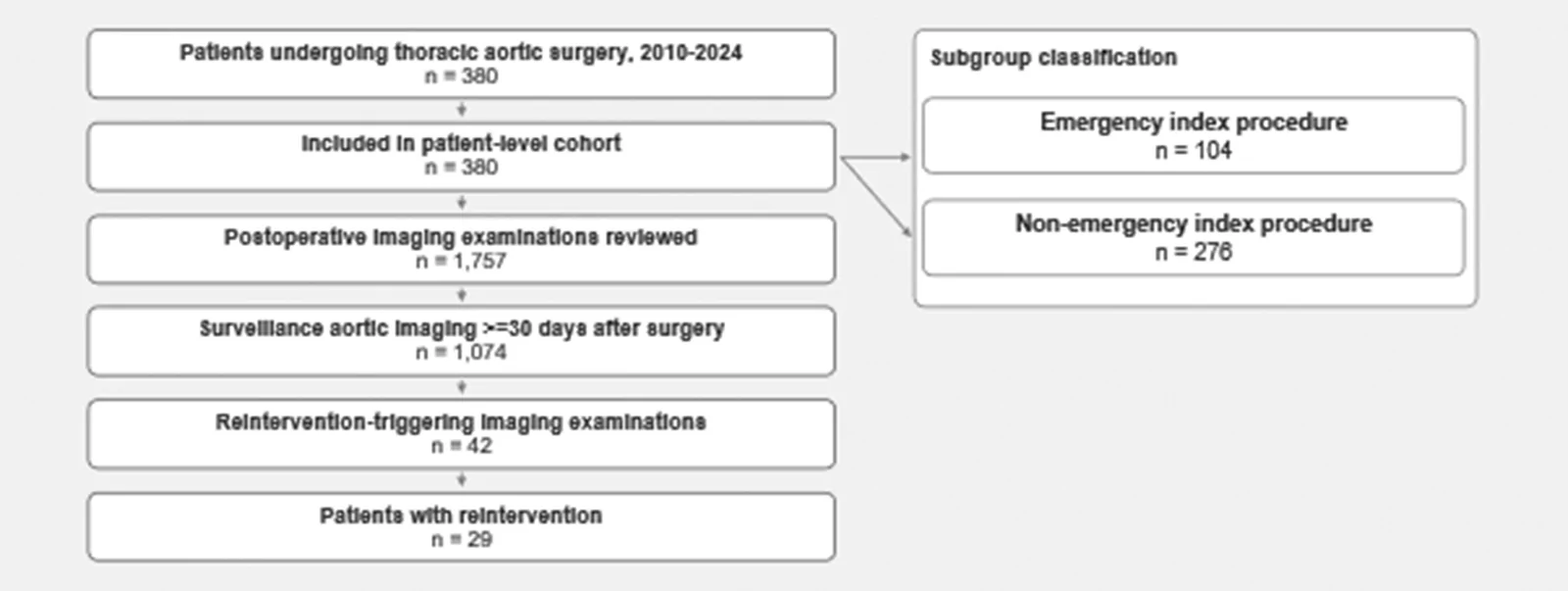
Cohort Flow Diagram Showing Formation of the Study Cohort and Imaging-Level Analysis Population

**Table 1.** Baseline Characteristics and Index Procedure Components of the Study Cohort

	Overall (*N* = 380)
Age at surgery, years, median (IQR)	63.0 (50.0-71.0)
Male sex, *n* (%)	288 (75.8%)
EuroSCORE II, median (IQR)	4.34 (2.13-9.85)
Follow-up, years, median (IQR)	6.07 (3.01-9.47)
Death during follow-up, *n* (%)	75 (19.7%)
Primary indication for index surgery, *n* (%)	
Elective aneurysm	265 (69.7%)
Dissection	88 (23.2%)
Rupture	9 (2.4%)
Missing	18 (4.7%)
Urgency of index procedure, *n* (%)	
Elective	250 (65.8%)
Urgent/emergency	130 (34.2%)
Index procedure components, *n* (%)^a^	
Supracoronary ascending aortic replacement	136 (35.8%)
Aortic root replacement or repair	184 (48.4%)
Aortic valve replacement	54 (14.2%)
Aortic arch repair/reconstruction	61 (16.1%)
Open descending thoracic aortic replacement	2 (0.5%)
TEVAR or descending thoracic endovascular stent graft implantation^b^	26 (6.8%)
CABG^c^	36 (9.5%)
Other valve procedure	26 (6.8%)
Comorbidities and clinical profile, *n* (%)	
Previous cardiac surgery	42 (11.1%)
Prior PCI^d^	3 (0.8%)
Coronary artery disease	28 (7.4%)
Diabetes requiring insulin	13 (3.4%)
Renal impairment, moderate or worse	97 (25.5%)
Dialysis	20 (5.3%)
Chronic lung disease	29 (7.6%)
Pulmonary hypertension, moderate/severe	18 (4.7%)
LV dysfunction, moderate or worse^e^	66 (17.4%)
NYHA class III-IV^f^	114 (30.0%)
CCS class 4 angina^g^	33 (8.7%)
Recent myocardial infarction	1 (0.3%)
Extracardiac arteriopathy	47 (12.4%)
Poor mobility	18 (4.7%)
Critical preoperative state	45 (11.8%)
Active endocarditis	4 (1.1%)
Bicuspid aortic valve	10 (2.6%)

a Procedure components are based on available procedure codes and operative registry data. Categories represent broad procedural components rather than detailed anatomical or device-specific classifications.

b Thoracic Endovascular Aortic Repair.

c Coronary Artery Bypass Graft.

d Percutaneous Coronary Intervention.

e Left Ventricular.

f New York Heart Association.

g Canadian Cardiovascular Society.

A total of 1074 imaging examinations were classified as surveillance aortic imaging performed ≥30 days after surgery. Overall, 42 imaging examinations led to reintervention, corresponding to 29 patients (7.6%), including 13 examinations leading to aortic reoperation in 12 patients. To contextualize imaging burden, yield was also expressed as the number of examinations performed per event. Overall, 41.8 examinations were performed per reintervention and 135 per aortic reoperation. When restricted to surveillance aortic imaging, 26.2 examinations were performed per reintervention and 82.6 per aortic reoperation (**Table 2**). Among surveillance aortic examinations, 3.8% resulted in reintervention. In addition, 18 examinations (1.0%) met treatment thresholds but were managed without immediate intervention; among surveillance aortic imaging, this occurred in 16 examinations (1.5%). Thus, directly or potentially actionable findings occurred in 60 examinations overall (3.4%) and in 57 surveillance aortic examinations (5.3%).

**Table 2.** Structure and Yield of Postoperative Imaging Surveillance

Metric	All postoperative imaging	Surveillance aortic imaging
Number of imaging studies	1757	1074
Reintervention-triggering images	42 (2.4%)	41 (3.8%)
Aortic reoperation-triggering images	13 (0.7%)	13 (1.2%)
Treatment threshold met, no immediate intervention	18 (1.0%)	16 (1.5%)
Directly or potentially actionable findings	60 (3.4%)	57 (5.3%)
Images per any reintervention	41.8	26.2
Images per aortic reoperation	135.0	82.6

Data are presented as *n* (%) unless otherwise indicated. Directly or potentially actionable findings include examinations leading directly to reintervention and examinations meeting treatment thresholds but managed without immediate intervention.

The distribution of reintervention types is shown in **Table 3**. The most common reinterventions after the index open thoracic aortic operation were open aortic reoperation for progressive dilatation (13 patients, 31.0%) and secondary Thoracic Endovascular Aortic Repair (TEVAR) (12 patients, 28.6%), followed by other secondary endovascular interventions (7 patients, 16.7%) and endoleak-related procedures after prior endovascular repair (4 patients, 9.5%).

**Table 3.** Mode of Detection and Type of Reintervention

Panel A. Detection context of reinterventions		
	Any reintervention** (*n* = 42)	Aortic reoperation*** (*n* = 13)
Elective surveillance image	30 (71.4%)	10 (76.9%)
Non-elective/urgent image	12 (28.6%)	3 (23.1%)

Panel B. Type of reintervention	
	*n* (%)
Open reoperation for aortic dilatation progression	13 (31.0%)
Secondary TEVAR after index open repair^a^	12 (28.6%)
Other secondary endovascular treatment for branch aneurysm/occlusion/leak	7 (16.7%)
Endoleak treatment after prior secondary endovascular repair	4 (9.5%)
Prosthetic infection/endocarditis	3 (7.1%)
Acute aortic pathology	2 (4.8%)
Access complication	1 (2.4%)

** Open reoperations and endovascular treatments.

*** Only open reoperations.

a Thoracic Endovascular Aortic Repair.

The temporal distribution of imaging yield demonstrated consistently low event rates across follow-up (**Table 4**, **Figure 3**). Reintervention occurred after 0.4% of examinations within 30 days of surgery and between 2.0% and 3.8% of examinations beyond 30 days. At the patient level, first reintervention most frequently occurred between 30 days and 3 years after surgery, although events were observed throughout follow-up. Median time to first reintervention was approximately 1.8 years. Kaplan-Meier analysis showed a cumulative incidence of reintervention of 2.8% at 1 year, 5.9% at 3 years, and 8.3% at 5 years (**Figure 2**). Death without prior reintervention occurred across all time intervals and represented a competing outcome.

**Figure 2.**
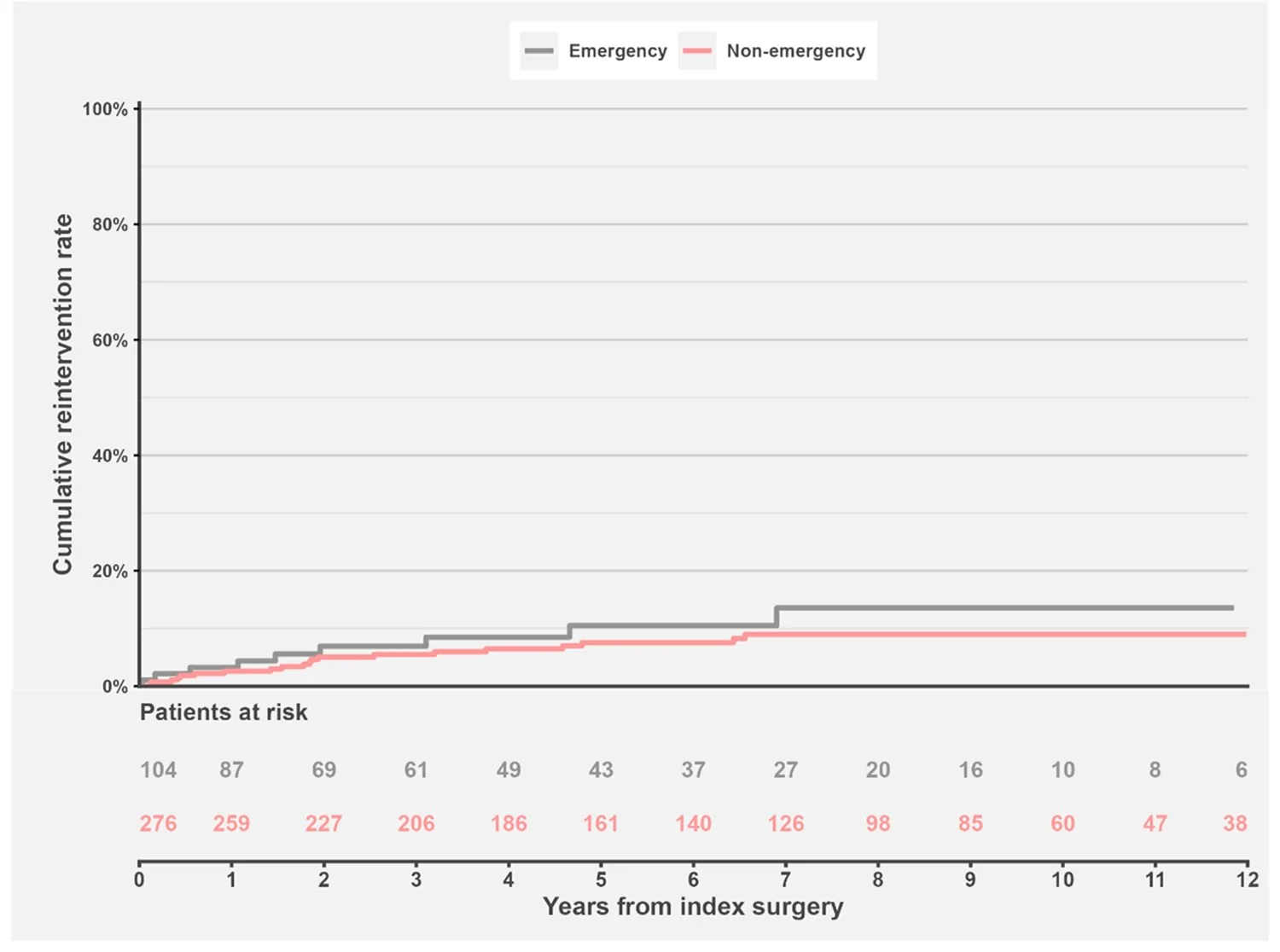
Cumulative Reintervention Rate After Index Thoracic Aortic Surgery Stratified by the Urgency of the Surgery. Kaplan–Meier curve showing time to first reintervention after index surgery. The numbers below the x-axis indicate patients at risk at each yearly time point

**Figure 3.**
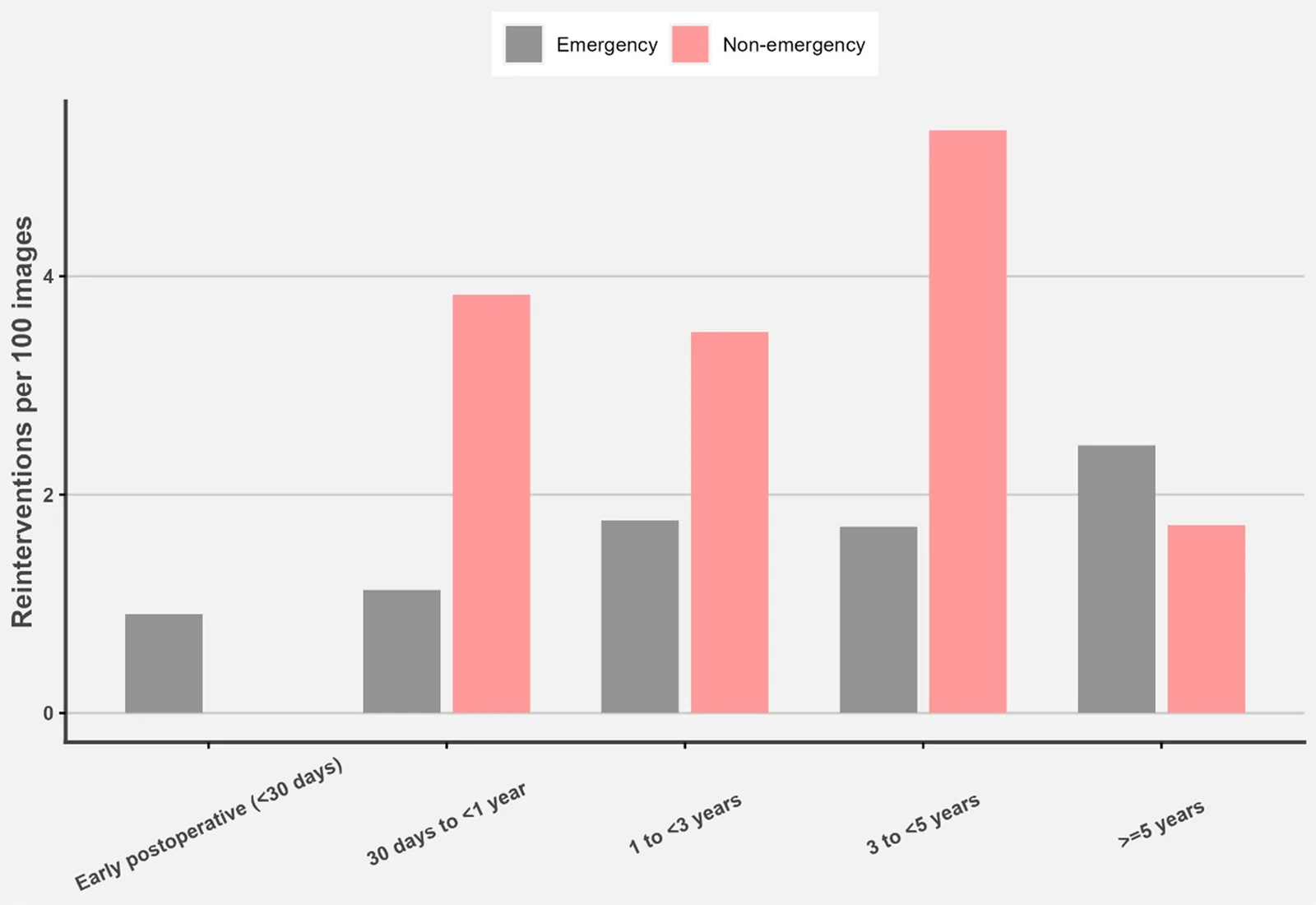
Yield of Postoperative Imaging According to Time from Index Surgery. Bars show the proportion of imaging studies that led to reintervention within each postoperative time interval. Numbers above bars indicate reintervention-triggering images divided by all images in that interval

**Table 4.** Timing, Intensity, and Discontinuation of Surveillance

Panel A. Reintervention yield according to time from index surgery				
Time from surgery	Imaging studies, *n*	Reintervention-triggering images, *n*	Aortic reoperation-triggering images, *n*	Reinterventions per image, **%**
<30 days	255	1	0	0.4
30 days to <1 year	412	11	2	2.7
1 to <3 years	428	12	3	2.8
3 to <5 years	267	10	4	3.7
≥5years	395	8	4	2.0

Panel B. Imaging intensity according to patient-level reintervention status		
	No reintervention (*n* = 351)	Reintervention (*n* = 29)
Median age at surgery, years	63.0 (50.5-71.0)	59.0 (48.0-68.0)
Median EuroSCORE II	4.17	7.44
Median all images per patient	2	11
Median surveillance-phase images per patient	2	10
Median surveillance aortic images per year	0.323	1.30

Panel C. Reasons for surveillance discontinuation or non-escalation among patients without reintervention (*n* = 351)	
Follow-up continued until censoring/no documented stop	239 (68.1%)
Only early postoperative imaging	50 (14.2%)
Death during follow-up	38 (10.8%)
Documented stop due to age/health	24 (6.8%)

Imaging intensity was 0.71 examinations per patient-year overall and 0.44 surveillance aortic examinations per patient-year. Reintervention and aortic reoperation rates were 0.017 and 0.0053 per patient-year, respectively. Patients undergoing reintervention had higher imaging intensity than those without reintervention (median 2.36 vs 0.53 examinations per patient-year; surveillance imaging 1.30 vs 0.32 per patient-year) (**Table 4**). Most reinterventions were identified during elective surveillance imaging (30/42, 71.4%), whereas 12 (28.6%) were detected during non-elective or urgent imaging (**Table 3**). For aortic reoperations, the corresponding proportions were 76.9% and 23.1%, respectively.

Follow-up patterns showed that 262 patients (68.9%) continued imaging until censoring without documented discontinuation, whereas 50 (13.2%) had only early postoperative imaging, 42 (11.1%) died during follow-up, and 26 (6.8%) had surveillance discontinued due to age or health status (**Table 4**).

In subgroup analysis, reintervention rates were similar between emergency and non-emergency patients (8.7% vs 7.3%, *P* = .67), with no significant difference in aortic reoperation (5.8% vs 2.2%, *P* = .10). Emergency patients underwent more frequent imaging (*P* < .001), but imaging yield remained comparable, with reintervention occurring after 1.6% vs 2.9% of examinations (*P* = .08). Most reinterventions were detected during elective surveillance (100% vs 60%). Five-year cumulative reintervention rates were 10.5% and 7.6%, respectively, and emergency status was not independently associated with reintervention (OR 0.72, 95% CI, 0.22-2.07).

## DISCUSSION

In this real-world cohort of patients undergoing thoracic aortic surgery, postoperative imaging surveillance was associated with substantial imaging burden but low overall diagnostic yield. Although most reinterventions were detected during elective surveillance, only 3.8% of surveillance aortic imaging examinations led to reintervention, corresponding to approximately 1 reintervention per 26 surveillance examinations and 1 aortic reoperation per 83 surveillance examinations. Reinterventions were concentrated in a small subset of patients, whereas most patients underwent repeated imaging without subsequent intervention.

These findings highlight an important limitation of current postoperative surveillance strategies. Contemporary American Heart Association/American College of Cardiology and European Society of Cardiology guidelines recommend regular imaging after thoracic aortic repair, with intensified surveillance in the presence of residual aortopathy.^1^^,^^2^ However, these recommendations are largely based on expert consensus and limited observational evidence rather than quantitative assessments of imaging yield, efficiency, or patient selection. Our results suggest that surveillance imaging is clinically relevant, but that its benefit is unevenly distributed across the postoperative population. This uneven distribution is particularly important because postoperative thoracic aortic patients do not represent a single homogeneous risk group. Surveillance requirements are likely to differ between patients with isolated proximal degenerative aneurysm repair, residual chronic dissection, distal thoracic pathology, connective tissue disease, hybrid repair, or endovascular components. Therefore, the pooled yield estimates in the present study should be interpreted as population-level averages rather than as evidence that all subgroups derive similar benefit from identical surveillance schedules.

Importantly, low reintervention rates should not be interpreted as evidence that surveillance imaging has limited clinical value. Reintervention is a strict and easily measurable end-point, but postoperative imaging may also detect clinically relevant findings that do not require immediate intervention, including progressive aortic dilatation, pseudoaneurysms, graft-related complications, infection, or residual aortopathy. Therefore, the low rate of reintervention-triggering imaging should be interpreted as a measure of procedural yield rather than the full clinical value of surveillance.

Our findings are consistent with and extend recent observational studies addressing postoperative aortic surveillance. Chung et al^7^ reported that structured follow-up imaging after aortic surgery was associated with increased detection of clinically relevant findings and higher reintervention rates, but without a corresponding survival benefit. Similarly, Kletzer et al^8^ evaluated follow-up intervals after non-emergent aortic surgery and concluded that omitting the first 6-month follow-up could result in missed aortic events, particularly after endovascular repair. In that study, however, the number needed to harm was substantially higher after open surgery than after endovascular repair, supporting the concept that surveillance yield differs by procedure type and patient subgroup. Our study adds to this literature by quantifying the number of imaging examinations required to identify actionable findings after thoracic aortic surgery and by showing that yield remains low at the population level.

The distribution of reinterventions provides further support for more individualized surveillance. Patients who ultimately underwent reintervention had higher imaging intensity during follow-up, but this finding should be interpreted as a marker of clinical selection and surveillance-related detection rather than as evidence that imaging intensity independently predicts reintervention. In routine practice, patients perceived to be at higher risk or showing possible disease progression are more likely to undergo repeated imaging, which also increases the probability of detecting actionable pathology. Consistent with this interpretation, emergency patients underwent more frequent imaging, yet reintervention rates and imaging yield were similar to those of non-emergency patients, suggesting that procedural urgency alone is insufficient to guide surveillance intensity. The temporal distribution of events also argues against a simple uniform surveillance schedule: event rates remained low across all follow-up intervals, without a clearly defined high-yield period. Moreover, although most reinterventions were detected during elective surveillance, a substantial minority occurred in non-elective or urgent imaging settings, particularly in relation to infection or acute complications. Thus, structured surveillance remains important, but it cannot replace symptom-driven or clinically indicated imaging and may be most useful when tailored to patient- and pathology-specific risk.

The observed low absolute event rate after surgery is also important in the context of the natural history of thoracic aortic aneurysm. Prior studies have shown that the risk of adverse aortic events is strongly related to aortic diameter and growth rate rather than uniformly elevated across all patients.^9^ In our cohort, the yield of surveillance imaging for reintervention and aortic reoperation was numerically within the range of reported event risks for untreated aneurysms of clinically relevant size. These figures are not directly comparable because the populations and end-points differ; untreated aneurysm events include rupture and dissection, whereas postoperative reinterventions are often detected electively and managed in a controlled setting. Nevertheless, the comparison emphasizes that surgery does not eliminate future aortic risk, and that postoperative surveillance should be evaluated not only by whether it detects the need for reinterventions, but also by how efficiently it identifies clinically meaningful findings, including those that inform continued monitoring or elective treatment planning.

Taken together, these findings support a shift from uniform, protocol-driven one-size-fits-all surveillance strategies towards more individualized, risk-based imaging approaches that account for underlying pathology, residual aortic disease, and extent of repair. Rather than applying identical follow-up schedules to all patients, future strategies could prioritize patients at higher risk of disease progression or complications while reducing low-yield imaging in low-risk individuals. This is clinically relevant because repeated CT-based surveillance carries cumulative burdens beyond diagnostic yield, including ionizing radiation exposure, repeated contrast administration, healthcare costs, logistical burden, and patient anxiety related to follow-up imaging.^10–13^ Recent risk projections suggest that CT-related radiation exposure may contribute meaningfully to future cancer incidence at the population level, while studies of aortic computed tomography angiography (CTA) surveillance in Marfan syndrome demonstrate measurable cumulative radiation exposure and excess lifetime cancer risk.^10^^,^^11^ Contrast-associated risks are lower with modern agents than historically assumed, but repeated contrast exposure remains relevant in patients with impaired renal function.^12^ These considerations do not argue against clinically necessary imaging, and our data do not establish that less intensive surveillance would be safe. However, they reinforce the need to identify patients in whom imaging intensity can be reduced without compromising clinically meaningful detection, particularly younger patients, patients with renal impairment, and those with low estimated event risk.

Several limitations should be considered. First, the observational design precludes causal inference regarding imaging intensity and reintervention. Second, residual confounding may persist, particularly with respect to unmeasured anatomical features, residual aortopathy, graft extent, and surgeon-specific follow-up practices. Third, the study was conducted at a single centre, which may limit generalizability. Fourth, the cohort included a heterogeneous spectrum of thoracic aortic disease, including aneurysms, dissections, proximal and distal thoracic pathology, elective and emergency procedures, and both open and endovascular repair components. The likelihood of reintervention is closely related to disease extent, residual aortopathy, clinical presentation, operative strategy, and surgeon-specific decision-making. Surveillance needs and long-term risks therefore likely differ substantially across clinically relevant subgroups, particularly in patients with residual chronic dissection, connective tissue disease, hybrid repair, or TEVAR. Because only 29 patients experienced reintervention, robust subgroup-specific comparisons according to pathology type, disease extent, residual aortopathy, or operative strategy were not feasible. Consequently, the overall yield estimates should be interpreted as population-level averages and may not apply equally to all subgroups. Fifth, we could not reliably distinguish staged or planned reinterventions from unexpected or unplanned reinterventions. If some reinterventions were part of an anticipated staged treatment strategy, the present analysis may overestimate the yield of surveillance imaging for newly detected actionable pathology. In that case, the number of surveillance examinations required to identify 1 unexpected reintervention would be even higher than reported. Finally, although complete follow-up was available within the regional healthcare system, events occurring outside the catchment area, particularly emergency imaging or interventions, may not have been fully captured and could have led to minor underestimation of event rates.

## CONCLUSION

In conclusion, postoperative imaging after thoracic aortic surgery was associated with low overall yield but substantial imaging burden, with clinically relevant findings concentrated in a minority of patients. In our experience, these findings suggest that current surveillance strategies may benefit from improved patient-specific targeting, but they do not establish that less intensive imaging would be safe without prospective validation.

## Data Availability

The data underlying this article will be shared on reasonable request to the corresponding author.
